# Supplementary material for: Holistic engineering of cell-free systems through proteome-reprogramming synthetic circuits
Source: Nat Commun. 2020 Jun 19;11:3138. doi: 10.1038/s41467-020-16900-7 (PMC7305103; doi:10.1038/s41467-020-16900-7)
Supplement: Supplementary file 1 — Description of Additional Supplementary Files [file 41467_2020_16900_MOESM1_ESM.pdf]

Title: Supplementary Data 1

Description: Data from the proteomics study of the whole-cell extracts.
