# Supplementary material for: Holistic engineering of cell-free systems through proteome-reprogramming synthetic circuits
Source: Nat Commun. 2020 Jun 19;11:3138. doi: 10.1038/s41467-020-16900-7 (PMC7305103; doi:10.1038/s41467-020-16900-7)
Supplement: Supplementary file 2 — Supplementary Information [file 41467_2020_16900_MOESM2_ESM.pdf]

Supplementary Information

Holistic engineering of cell-free systems through proteome-reprogramming synthetic circuits

Contreras-Llano, L.E., & Meyer, C., *et al.* 2020

Supplementary Note 1. Analysis of protein expression through deGFP quantification

To measure the protein expression of our multi-strain cell lysates and controls, we designed a vector with the gene encoding deGFP under the control of the T7 promoter. Different versions of this plasmid were built and tested to find the vector that yielded the highest deGFP expression in our system (Methods, Section M1). For the quantification of deGFP produced by our *E. coli* cell-lysates, we constructed a calibration curve using purified eGFP (BioVision, Inc) as a standard (Supplementary Fig. 6, Methods, Section M4).

Supplementary Note 2. Optimization of cell lysate preparation and reaction buffer

The whole-cell lysate preparation protocol was optimized by varying pellet to sonication buffer ratio, energy input for the sonication of the cellular pellet, and runoff duration. Different growth media were tested following the same whole-cell lysate preparation protocol, and the media that showed an advantage over the others was incorporated into the protocol in all subsequent whole cell lysate preparations (Supplementary Fig. 2). The conditions for CFPS were optimized by varying template DNA concentration, expression time, temperature, and agitation speed during incubation (Supplementary Fig. 1). The optimized protocol for obtaining our multi-strain cell lysates and for the assembly of the CFPS reactions is detailed in Methods, Section M2.

The optimized reaction buffer used in this study is a modification of the S12 supplement described in our previous work [1]. Briefly, the S12 supplement used as an starting point for our optimized reaction buffer consists of the following: 50 mM HEPES (pH 7.6), 1.2 mM rATP, 0.8 mM rGTP/rCTP/rUTP, 0.17 mg mL^{-1} tRNA, 34 μg mL^{-1} Folinic acid, 12 mM Magnesium acetate, 50 mM Potassium gluconate, 80 mM Ammonium acetate, 2% PEG 8,000, 2 mM DTT, 4 mM Spermidine, 80 μg mL^{-1} Creatine kinase, 67 mM Creatine phosphate, 0.64 mM cAMP, 1.5 mM of

each 20 amino acids. The use of our optimized reaction buffer (Methods, Section M3.) resulted in ~2-fold more deGFP expressed compared to the S12 supplement control (Supplementary Fig. 4).

Supplementary Note 3. SDS-PAGE analysis of CFPS reactions supplemented with purified translation machinery

To confirm that the translation machinery protein concentrations supplemented are comparable to those present in BL-18S_{WCE} and BL-7S_{WCE}, we analyzed and compared the reactions in Fig. 2B&C with control reactions through SDS-PAGE (Supplementary Fig. 7). The change in band intensities shows the staged increase in translation machinery proteins in the reactions supplemented with the purified mixtures. During gel analysis, it is noticeable that we can closely match and surpass the concentrations of the overexpressed proteins in BL-18S_{WCE} and BL-7S_{WCE}.

Supplementary Note 4. Proteomics analysis of cell lysates

All data used for the proteomics analysis are included in the Supplementary Data 1. This file includes the original and normalized data and their gene ontological function assignments.

To allow for quantitative comparisons of the proteins between the individual samples used for TMT-mass spectrometry, we also labelled and processed pooled samples comprised of equal portions of each extract to allow for internal reference scaling (IRS) normalization (Methods, Section M7). The comparison of each extract and the effects of the normalization process are depicted in Supplementary Fig. 8. The principal component analysis of all replicates of each extract is shown in Fig. 3B. There is clear clustering of the replicates, indicating that they are representative without outliers.

There is a prominent difference between the intensities of the intentionally overexpressed proteins across the samples, as seen in Fig. 3C indicated in black. The intended enrichment of CFP and EF-

Tu/EF-Ts is clearly observed in the BL-CFP_{WCE} and BL-$S1_{WCE}$, respectively. All over-expressed proteins in BL-$7S_{WCE}$ were enriched by more than 2-fold with some as high as 12-fold, except for RF-1 and RF-2, which showed no significant difference. This result was anticipated because the strain expressing those proteins comprise ~1% of the total inoculum. There is also an 82% and 67% increase in the intensity of EF-Tu and EF-Ts, respectively, when comparing BL-$S1_{WCE}$ to BL-$7S_{WCE}$, as would be expected by the dilution of Strain-1 in BL-$7S_{WCE}$.

Supplementary Table 1

Strain	Gene 1<br>pIURAH backbone	Gene 2<br>pIURKL backbone	BL-18S	BL-7S	BL-8S
			Relative density %		
**1**	EF-Tu	EF-Ts	55.49	57.80	46.24-28.90
**2**	IF1	IF2	15.95	16.61	13.29-8.31
**3**	EF-G	IF3	3.60	3.75	3.00-1.88
**4**	Ala-tRNA transferase	EF4	7.97	8.30	6.64-4.15
**5**	RF1	RF2	0.48	0.50	0.40-0.25
**6**	RF3	RRF	4.91	5.12	4.10-2.56
**7**	EF-G	---	7.59	7.91	6.33-3.96
**8**	Leu-tRNA transferase	Met-tRNA formyltransferase	0.63	---	---
**9**	His-tRNA transferase	Glu-tRNA transferase	0.57	---	---
**10**	Phe-tRNA transferase A	Lys-tRNA transferase	0.44	---	---
**11**	Pro-tRNA transferase	Val-tRNA transferase	0.37	---	---
**12**	Met-tRNA transferase	Asp-tRNA transferase	0.52	---	---
**13**	Ile-tRNA transferase	Gln-tRNA transferase	0.48	---	---
**14**	Phe-tRNA transferase B	Trp-tRNA transferase	0.45	---	---
**15**	Asn-tRNA transferase	Ser-tRNA transferase	0.35	---	---
**16**	Tyr-tRNA transferase	Arg-tRNA transferase	0.09	---	---
**17**	Gly-tRNA transferase A	Cys-tRNA transferase	0.07	---	---
**18**	Gly-tRNA transferase B	Thr-tRNA transferase	0.03	---	---
**19**	Gam	---	---	---	20-50

Supplementary Table 1. Detailed strain composition of the 18-, 7- and 8-strain bacterium consortia.

Supplementary Figures

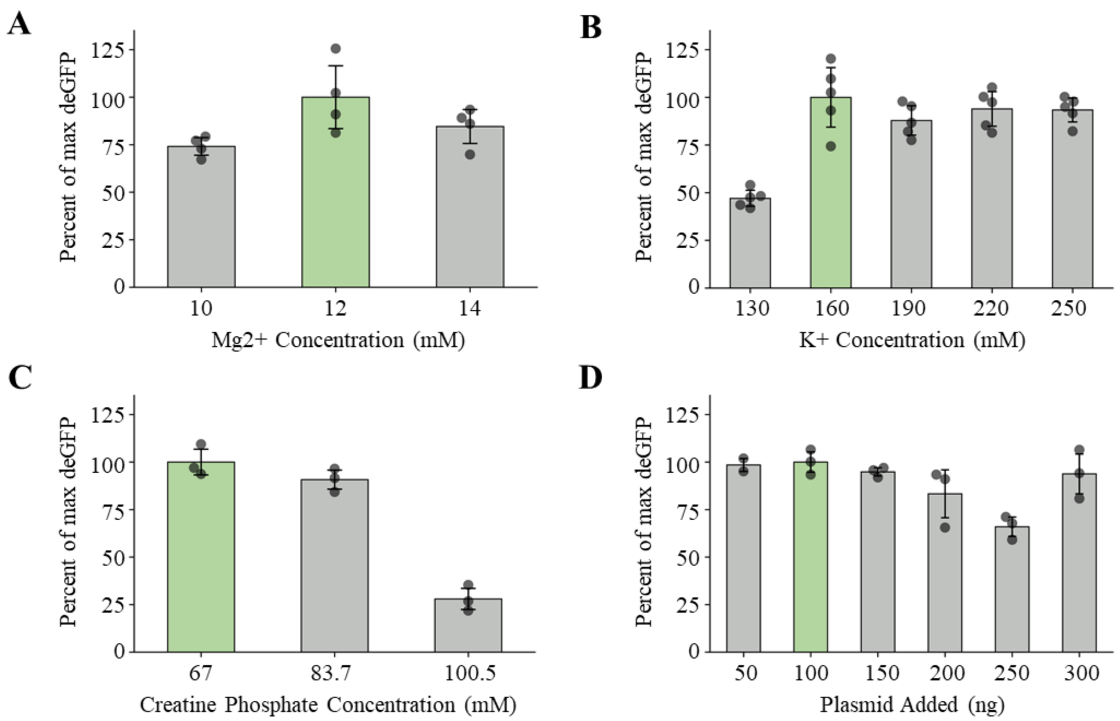

Supplementary Fig. 1. Optimization of cell-free reaction conditions

Reactions with a range of component concentrations were tested, varying only one component per experiment. The concentration with the highest yield was selected and used in subsequent reactions. A green colored bar indicates the chosen condition. Data were normalized based on the highest expression achieved within each component test. Data are presented as normalized mean values & error bars represent normalized standard deviation. **A)** Magnesium concentration (n=4 two independent reactions from two independent extract preparations), **B)** potassium concentration (n=5 two independent reactions of one extract preparation and three reactions of another), **C)** creatine phosphate (n=3 three independent reactions using one extract preparation), **D)** plasmid added (n=3 three independent reactions using one extract preparation). Source data are provided as a Source Data file.

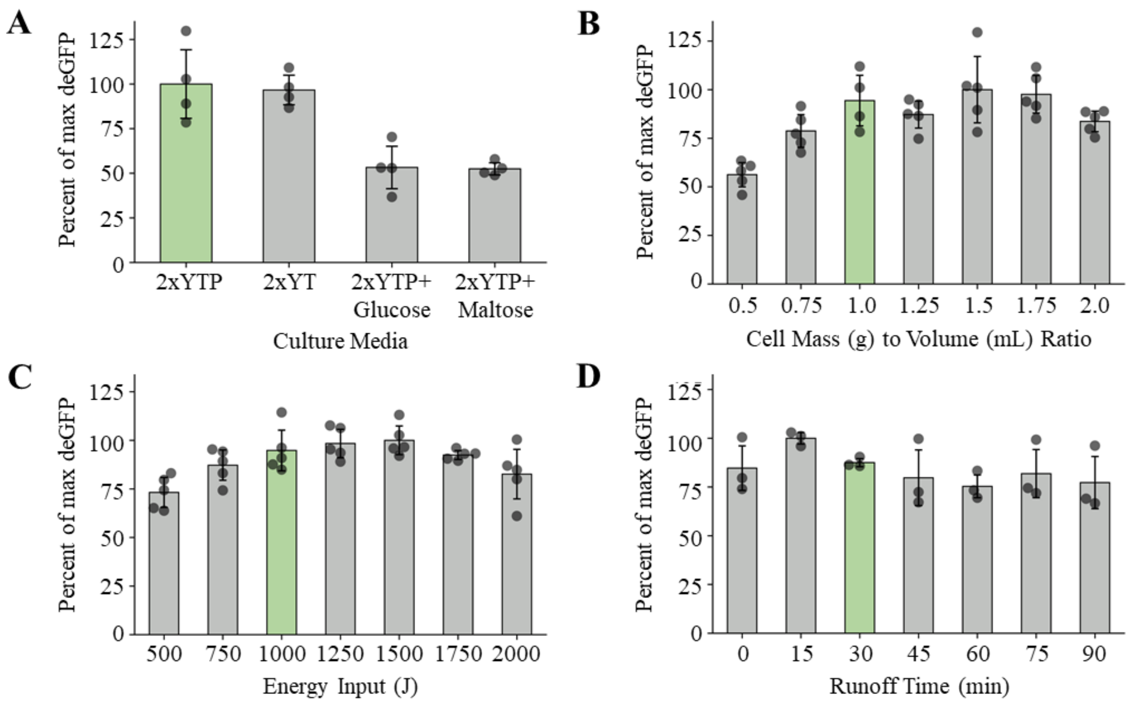

Supplementary Fig. 2. Optimization of whole-cell extract preparation

The preparation protocol of whole-cell extract was optimized in terms of the following parameters: **A)** Different growth media to culture the cells (n=4 two independent reactions from two independent extract preparations), **B)** The cell mass (grams) to buffer volume (mL) used to resuspend washed cells (n=4 two independent reactions from two independent extract preparations), **C)** the energy input during tip sonication (n=5 two independent reactions of one extract preparation and three reactions of another), **D)** The duration of time the extracts were incubated after sonication and centrifugation (n=3 three independent reactions from one extract preparation). A green colored bar indicates the chosen condition. Data were normalized based on the highest expression achieved within each component test. Data are presented as normalized mean values & error bars represent normalized standard deviation. Source data are provided as a Source Data file.

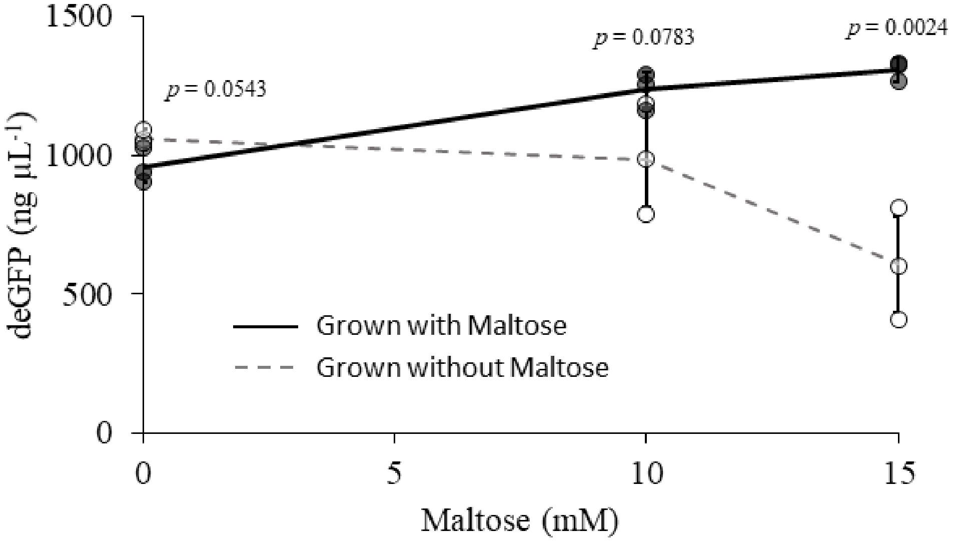

Supplementary Fig. 3. Addition of maltose to cell-free reactions

Maltose was added at various concentrations to stimulate inorganic phosphate utilization to maltose metabolism, as described previously [2]. Two different types of extracts were prepared to test this modification, one BL-7S consortium grown with maltose supplemented in the 2xYTP growth media (Black) and one without (Grey). The addition of maltose to the growth media was intended to increase the concentration of maltose phosphorylase, an enzyme critical for the metabolism of maltose. CFPS reactions were assembled with these lysates and variable amounts of maltose. The extract cultured with standard conditions showed a decrease in expression with increasing maltose. The standard deviation also increased with maltose. The extract grown with maltose showed a 36% increase in expression with 15 mM maltose. Extracts grown with maltose produced over 2-fold more protein than the extract without in the 15 mM maltose condition. Data are presented as mean values & each error bar represents normalized s.d. (n=3 independent experiments). Standard two-tail *t*-test. Source data are provided as a Source Data file.

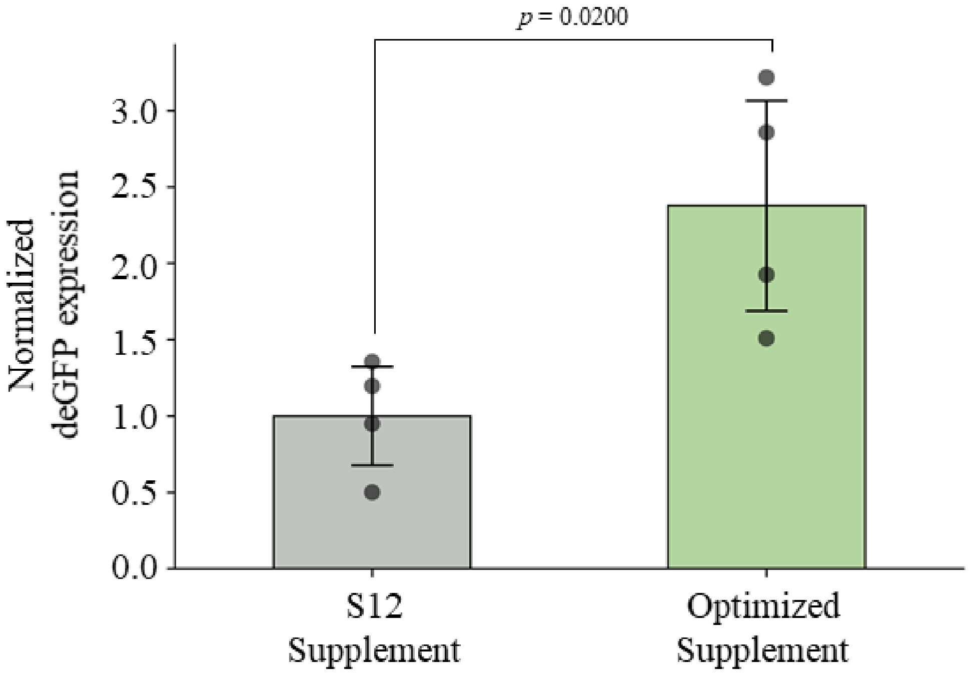

Supplementary Fig. 4. Comparison of deGFP expression between CFPS reactions assembled using S12 supplement and the optimized reaction buffer.

The expression of deGFP using conventional S12 buffer [1] and an optimized reaction buffer was assessed in reactions assembled using BL-7S$_{WCE}$. Reactions assembled using the optimized buffer showed ~2-fold more deGFP expression compared to reactions assembled using conventional S12 supplement. Data are presented as normalized mean values & each error bar represents normalized s.d. (n=4 independent experiments). Standard two-tail *t*-test. Source data are provided as a Source Data file.

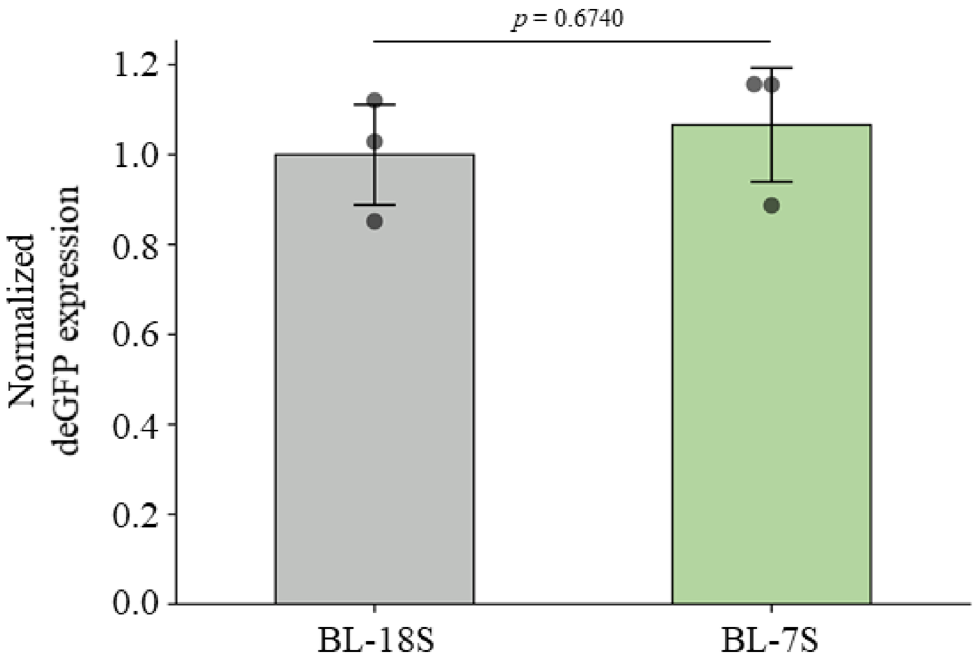

Supplementary Fig. 5. Comparison of deGFP expression level between CFPS reactions assembled using BL-7S$_{WCE}$ and BL-18S$_{WCE}$.

We generated 3 different batches of BL-7S$_{WCE}$ and BL-18S$_{WCE}$ and compared the deGFP expression achieved in reactions assembled using each one of the extracts. The results show that reactions assembled with BL-7S$_{WCE}$ and BL-18S$_{WCE}$ have a comparable expression level. Data are presented as normalized mean values & each error bar represents normalized s.d. (n=3 independent experiments). Standard two-tail *t*-test. Source data are provided as a Source Data file.

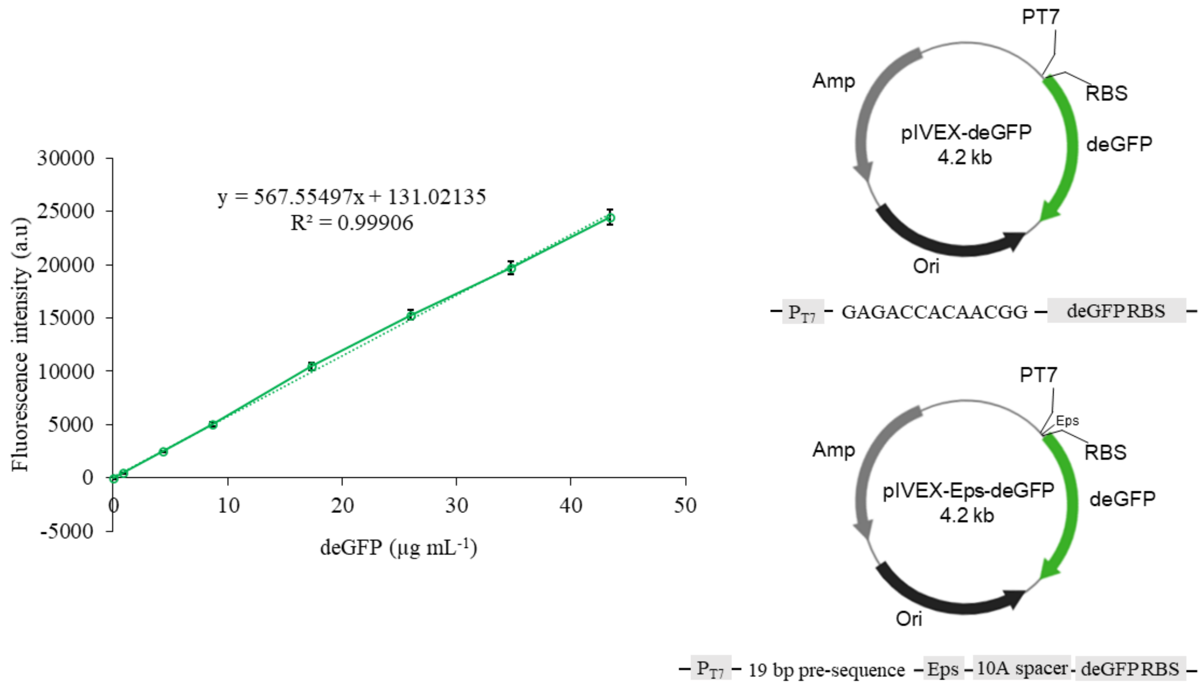

Supplementary Fig. 6. eGFP standard curve for deGFP quantification.

We built a standard curve for deGFP quantification by diluting pure eGFP (BioVision, Inc) using the dilution buffer specified in Methods, Section M4. Independent serial dilutions were made in triplicate, and the fluorescence intensity of each dilution was measured using NanoQuant plate (Tecan) and an m1000Pro Infinite plate reader. Excitation and emission wavelength used to measure the fluorescence of eGFP were 488 and 507nm, respectively. The individual values obtained from each dilution were plotted, and the linear equation from the fitted trendline was used to quantify deGFP expression through this study. Each error bar represents s.d. of the three independent serial dilutions. Plasmid maps show the plasmids used throughout this study for CFPS of deGFP. Source data are provided as a Source Data file.

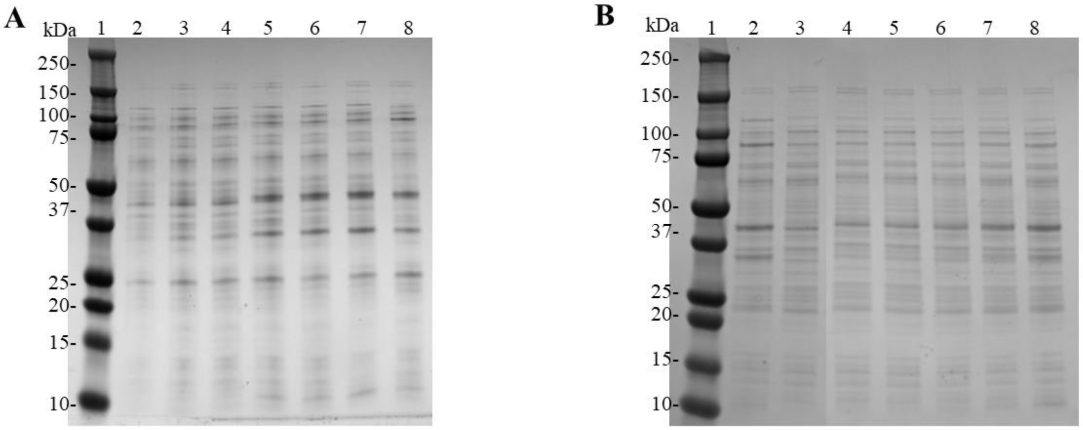

Supplementary Fig. 7. SDS-PAGE analysis of CFPS reactions supplemented with purified translation machinery.

SDS-PAGE analysis showing representative samples (n=1/4) from the experiments supplementing 12 (A) and 34 (B) translation machinery proteins to CFPS reactions assembled using BL-CFP$_{WCE}$ & BL-E$_{WCE,}$ respectively. The gels show the enrichment of translation machinery to comparable levels to those in cell lysates prepared using the BL-18S and BL-7S consortia. Supplementation of proteins was compared against reactions assembled using single strain controls (BL-CFP$_{WCE}$ & BL-E$_{WCE}$, Lanes 2 & 3 in gels A & B respectively). Translation machinery enrichment in all reactions was compared against reactions assembled using different consortia (BL-7S$_{WCE}$ & BL-18S$_{WCE}$, Lanes 8 & 2 in Gels A & B, respectively. Translation machinery concentration increases proportionally with the supplementation of up to 18 μg of purified translation machinery mixtures (Lanes 3 to 7, and 4 to 8 in gels A & B, respectively). All reactions and controls were diluted 1:40 for SDS-PAGE analysis.

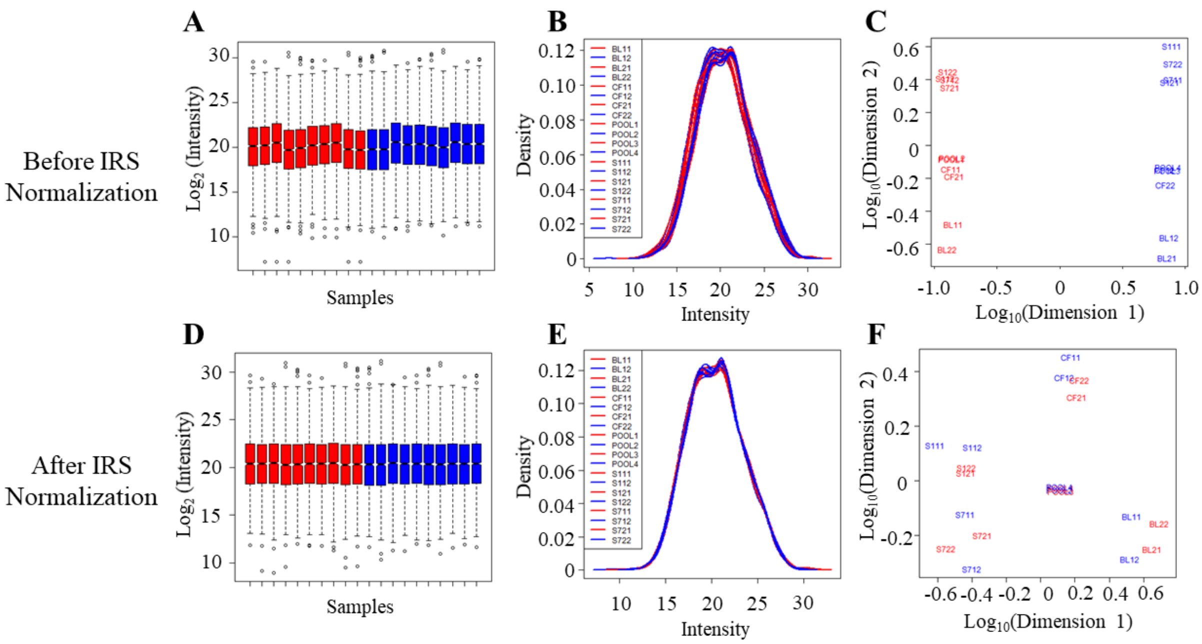

Supplementary Fig. 8. IRS normalization of TMT-mass spectrometry results.

A-C display the data prior to normalization, while **D-F** display the data post normalization. The two different 10plex samples are colored red and blue. (n=1852 different proteins). **A & D** show the boxplots representing the $\log_2$ of each protein intensity identified in each extract. Center line, median; box limits, 75th and 25th quartiles; whiskers, 1.5×interquartile range; points, outliers. After normalization, there is less deviation in the distribution of intensities. **B & E** show the density plots of protein intensities in each sample. After normalization, the traces are nearly identical. **C & F** display the principal component analysis of all the samples. Prior to normalization, there is a clear grouping of the samples based on the set of labels used to identify them. After normalization, the individual replicates group according to the extract. Source data are provided as a Source Data file.

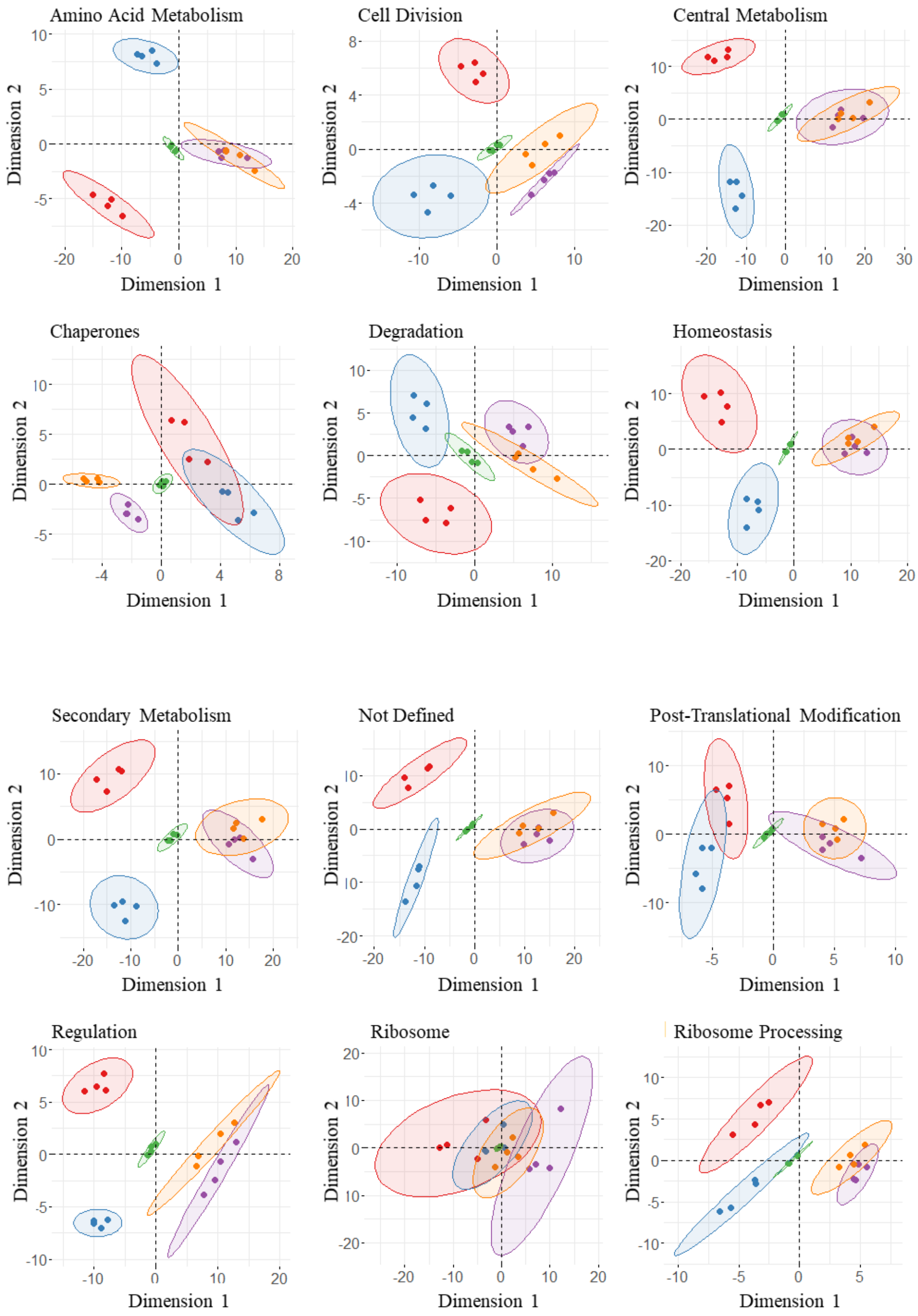
Amino Acid Metabolism
Cell Division
Central Metabolism
Chaperones
Degradation
Homeostasis
Secondary Metabolism
Not Defined
Post-Translational Modification
Regulation
Ribosome
Ribosome Processing
Dimension 1
Dimension 2

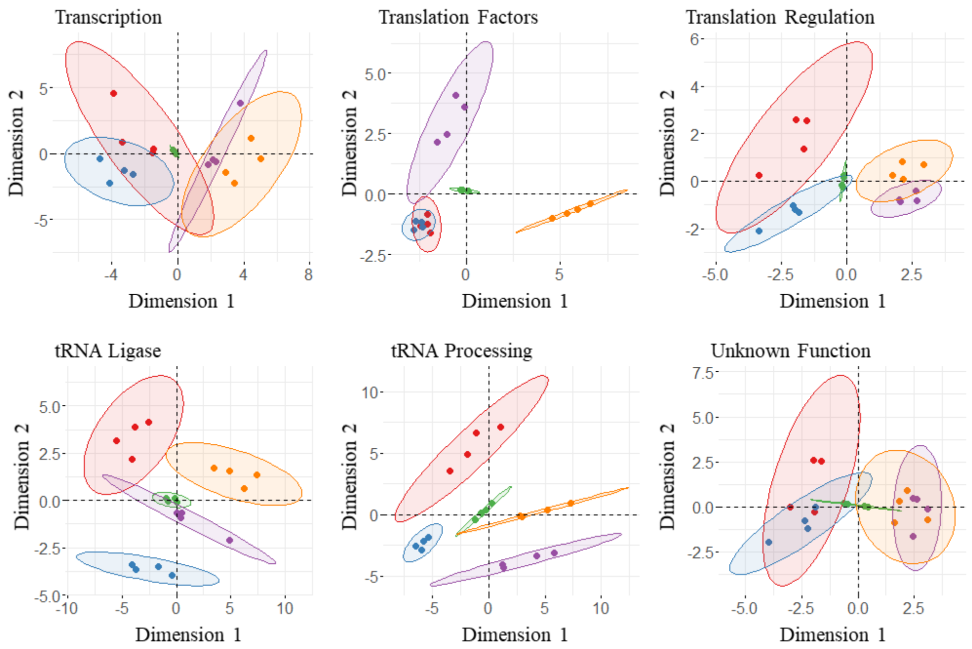

Supplementary Fig. 9. Principle component analysis of each protein category.

Principal component analysis was conducted on all replicates of each extract within each functional protein category. Ellipses indicate the boundary for statistical significance by a two-tailed t-distribution (n=4 two independent samples from two independent extract preparations). Red: BL-E, Blue: BL-CFP, Purple: BL-1S, Yellow: BL-7S, Green: Pooled. Source data are provided as a Source Data file.

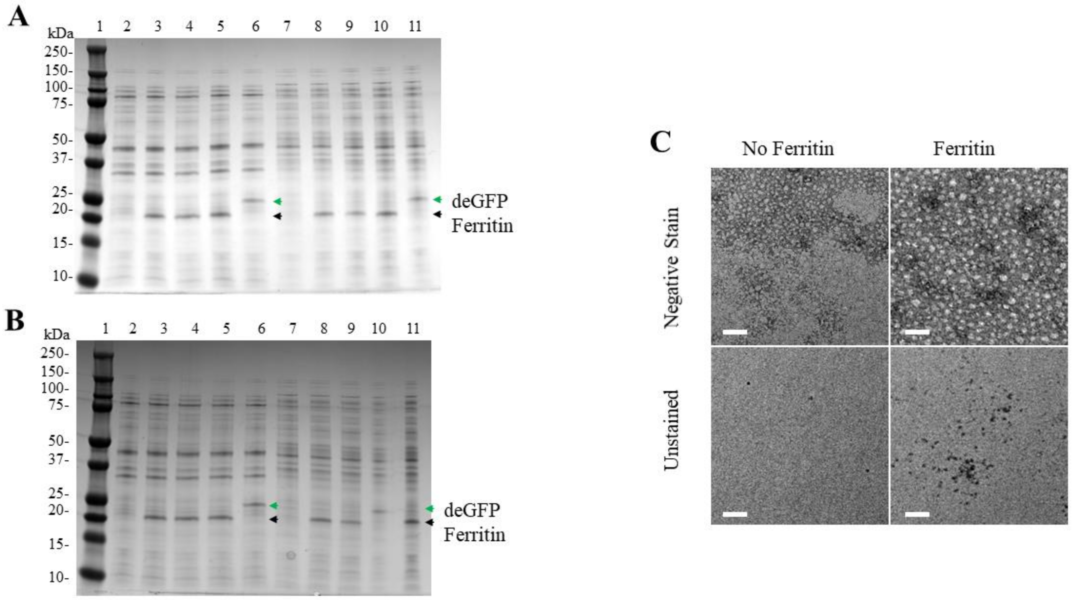

Supplementary Fig. 10. Analysis of CFPS reactions expressing ferritin.

A&B) SDS-PAGE analysis of CFPS reactions assembled with BL-7S$_{WCE}$ and BL-E$_{WCE}$ expressing ferritin. Lanes 3-5 & 8-10 in gel (**A**) and 3-5 & 8, 9 and 11 in gel (**B**) show three independent biological replicates of the reactions expressing ferritin with BL-7S$_{WCE}$ and BL-E$_{WCE}$ respectively. Expression was compared against negative control reactions without DNA (Lanes 2 and 7 in both gels), and positive controls expressing deGFP (Lanes 6 & 11 in gel (A) and 6 & 10 in gel (B)). All reactions and controls were diluted 1:40 for SDS-PAGE analysis. The two technical replicates used for the quantification of ferritin are shown. **C)** Transmission electron microscopy images of ferritin nanocages. The images on the top show the stained samples, while the images on the bottom show unstained samples. Left side images show CFPS samples with no ferritin expression as negative controls. The assembly of the ferritin nanocage and its imaging using transmission electron microscopy (See Methods, Section M9) was performed in triplicate (three independent experiments) with the three experiments showing the same results. The scale bar represents 100 nm.

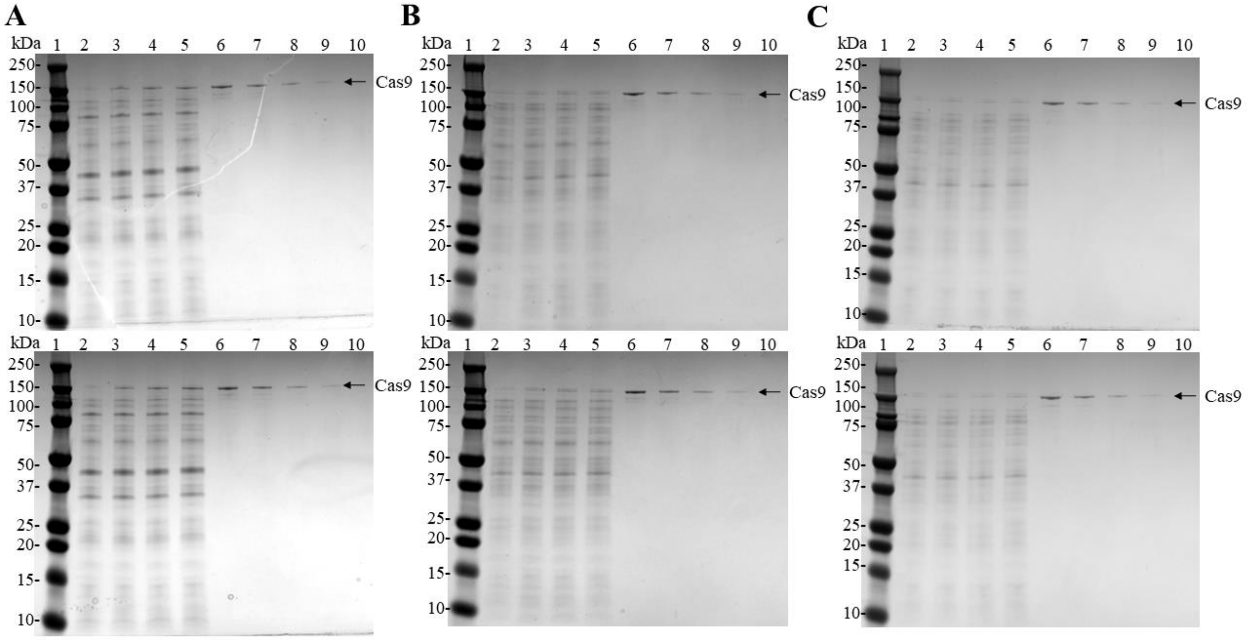

Supplementary Fig. 11. SDS-PAGE analysis of CFPS reactions expressing Cas9.

SDS-PAGE analysis of CFPS reactions assembled with BL-7S$_{WCE}$ **(A)**, BL-E$_{WCE}$ **(B),** and S30 **(C)** expressing Cas9. Lanes 3 to 5 in all gels shows three independent biological replicates of the reactions expressing Cas9. Expression was compared against negative control reactions without DNA (Lane 2 in all gels) and a gradient of purified Cas9 ranging from 1.61 mg mL^{-1} to 0.2 mg mL^{-1} (Lanes 6 to 9). All reactions, negative controls, and purified Cas9 were diluted 1:40 for SDS-PAGE analysis.

Supplementary References

1. Ding, Y., Contreras-Llano, L. E., Morris, E., Mao, M. & Tan, C. Minimizing Context Dependency of Gene Networks Using Artificial Cells. *ACS Appl. Mater. Interfaces* **10**, 30137–30146 (2018).
2. Caschera, F. & Noireaux, V. Synthesis of 2.3 mg/ml of protein with an all Escherichia coli cell-free transcription-translation system. *Biochimie* **99**, 162–168 (2014).
