# Supplementary material for: Holistic engineering of cell-free systems through proteome-reprogramming synthetic circuits
Source: Nat Commun. 2020 Jun 19;11:3138. doi: 10.1038/s41467-020-16900-7 (PMC7305103; doi:10.1038/s41467-020-16900-7)
Supplement: Supplementary file 4 — Reporting Summary [file 41467_2020_16900_MOESM4_ESM.pdf]

nature research

Corresponding author(s): Cheemeng Tan

Last updated by author(s): May 19, 2020

Reporting Summary

Nature Research wishes to improve the reproducibility of the work that we publish. This form provides structure for consistency and transparency in reporting. For further information on Nature Research policies, see Authors & Referees and the Editorial Policy Checklist .

Statistics

For all statistical analyses, confirm that the following items are present in the figure legend, table legend, main text, or Methods section.

n/a	Confirmed	
☐	☒	The exact sample size (*n*) for each experimental group/condition, given as a discrete number and unit of measurement
☐	☒	A statement on whether measurements were taken from distinct samples or whether the same sample was measured repeatedly
☐	☒	The statistical test(s) used AND whether they are one- or two-sided<br>*Only common tests should be described solely by name; describe more complex techniques in the Methods section.*
☒	☐	A description of all covariates tested
☐	☒	A description of any assumptions or corrections, such as tests of normality and adjustment for multiple comparisons
☐	☒	A full description of the statistical parameters including central tendency (e.g. means) or other basic estimates (e.g. regression coefficient) AND variation (e.g. standard deviation) or associated estimates of uncertainty (e.g. confidence intervals)
☐	☒	For null hypothesis testing, the test statistic (e.g. *F*, *t*, *r*) with confidence intervals, effect sizes, degrees of freedom and *P* value noted<br>*Give P values as exact values whenever suitable.*
☒	☐	For Bayesian analysis, information on the choice of priors and Markov chain Monte Carlo settings
☒	☐	For hierarchical and complex designs, identification of the appropriate level for tests and full reporting of outcomes
☒	☐	Estimates of effect sizes (e.g. Cohen's *d*, Pearson's *r*), indicating how they were calculated

Our web collection on statistics for biologists contains articles on many of the points above.

Software and code

Policy information about availability of computer code

Data collection	Data collection performed using Excel for Office 365, Tecan i-control (Version 3.9.1.0), GeneTools (Version 4.03.05.0)
Data analysis	Data analysis performed using Excel for Office 365, Fiji (Image J Version 1.51v), The PAW pipeline (Version 1), Python 3.6 and R 3.6.1. All custom code used to interpret and analyze the protein abundances was deposited in GitHub and its publicly available at [https://github.com/ccmeyer/TMT-analysis].

For manuscripts utilizing custom algorithms or software that are central to the research but not yet described in published literature, software must be made available to editors/reviewers. We strongly encourage code deposition in a community repository (e.g. GitHub). See the Nature Research guidelines for submitting code & software for further information.

Data

Policy information about availability of data

All manuscripts must include a data availability statement. This statement should provide the following information, where applicable:

- Accession codes, unique identifiers, or web links for publicly available datasets
- A list of figures that have associated raw data
- A description of any restrictions on data availability

All data used to generate Figs. 1B-D, 2B&C, 3A-E, 4A, C&D, and Supplementary Figs. 1A-D, 2A-D, 3, 4, 5, 6, 8 & 9 in this manuscript are included as a Source Data File. The identified protein abundances and internal reference normalized data from the proteomics study are provided in Supplementary Data 1. This file also includes the specifications for gene ontological assignments of each protein. The mass spectrometry proteomics data have been deposited to the ProteomeXchange Consortium via the PRIDE partner repository with the dataset identifier PXD018858 [https://www.ebi.ac.uk/pride/archive/projects/PXD018858].

Field-specific reporting

Please select the one below that is the best fit for your research. If you are not sure, read the appropriate sections before making your selection.

☒ Life sciences ☐ Behavioural & social sciences ☐ Ecological, evolutionary & environmental sciences

For a reference copy of the document with all sections, see nature.com/documents/nr-reporting-summary-flat.pdf

Life sciences study design

All studies must disclose on these points even when the disclosure is negative.

Sample size	Sample size (n=3 independent experiments or higher) was chosen based on what is a typical sample size for cell-free protein synthesis studies as described in literature. See Borkowski, O., Bricio, C., Murgiano, M. et al. Cell-free prediction of protein expression costs for growing cells. Nat Commun 9, 1457 (2018). [https://doi.org/10.1038/s41467-018-03970-x]., Villarreal, F., Contreras-Llano, L., Chavez, M. et al. Synthetic microbial consortia enable rapid assembly of pure translation machinery. Nat Chem Biol 14, 29–35 (2018). [https://doi.org/10.1038/nchembio.2514], Ding, Y., Contreras-Llano, L. E., Morris, E., Mao, M. & Tan, C. Minimizing Context Dependency of Gene Networks Using Artificial Cells. ACS Appl. Mater. Interfaces 10, 30137–30146 (2018). [https://doi.org/10.1021/acsami.8b10029], & Marshall, R., et al. Rapid and Scalable Characterization of CRISPR Technologies Using an E. coli Cell-Free Transcription-Translation System. Molecular Cell 69, 1 146-157 (2018) [https://doi.org/10.1016/j.molcel.2017.12.007].
Data exclusions	The semi-continuous exchange data using the commercial S30 extract was excluded as the protocol used was not defined by the manufacturer and therefore its poor performance was not considered a fair representation of its capacity.
Replication	For all experiments at least 3 independent experiments were performed (with multiple technical replicates each) and were used for statistical analyses. All raw and analyzed data collected from these experiments is included as a Source Data file and all attempts of replication were successful.
Randomization	Neither randomization or group allocation were necessary for our experiments since samples were organized by experimental variables, characterized, and reported in full.
Blinding	Blinding was not necessary since control groups were included in all experiments.

Reporting for specific materials, systems and methods

We require information from authors about some types of materials, experimental systems and methods used in many studies. Here, indicate whether each material, system or method listed is relevant to your study. If you are not sure if a list item applies to your research, read the appropriate section before selecting a response.

Materials & experimental systems

n/a	Involved in the study
☒	☐ Antibodies
☒	☐ Eukaryotic cell lines
☒	☐ Palaeontology
☒	☐ Animals and other organisms
☒	☐ Human research participants
☒	☐ Clinical data

Methods

n/a	Involved in the study
☒	☐ ChIP-seq
☒	☐ Flow cytometry
☒	☐ MRI-based neuroimaging
